# Diseases of the Prostate

**Published:** 1886-12

**Authors:** 


*Diseases of the Prostate.* By Sir HENRY THOMPSON. Pp. 237. Sixth Edition. London: J. & A. CHURCHILL.

A notice of a sixth edition of this work is almost superfluous. Since it was awarded the prize as Jacksonian Essay in 1860, it has remained the English classic on the subject which it deals with. In this edition some modifications and additions have been made in theory and in practice, while a novel classification of prostatic deposits has been proposed.

We confess that we should have liked to have seen more notice taken of the work of certain provincial surgeons who have made reputations for themselves in the treatment of prostatic diseases. It is just possible that, in the light of Sir Henry Thompson's extensive experience, the work of these men is considered valueless; but many men of repute do not think so, and it would have at least given a thoroughness and finish to the work which it does not possess had their labours been referred to. On the other hand, we cannot help noting a tendency to play the foster-father to the offspring of other brains; it is satisfactory that the depth of his affection for such offspring seems to be in direct ratio with the amount of neglect which the real parent has exhibited.

